# Positive Lymph Nodes Independently Affect Long-Term Survival After Pancreaticoduodenectomy for Non-Ampullary Duodenal Adenocarcinoma: A Single-Center, Retrospective Analysis

**DOI:** 10.3390/jcm14082616

**Published:** 2025-04-11

**Authors:** Matteo De Pastena, Caterina Costanza Zingaretti, Salvatore Paiella, Gabriella Lionetto, Massimo Guerriero, Nicoletta De Santis, Claudio Luchini, Giuseppe Malleo, Roberto Salvia

**Affiliations:** 1 Pancreatic Surgery Unit, University of Verona Hospital Trust, 37126 Verona, Italy; 2 University of Verona, 37129 Verona, Italy; 3 Pancreatic Surgery Unit, Department of Surgery, Dentistry, Paediatrics and Gynaecology, University of Verona, 37129 Verona, Italy; 4 Clinical Research Unit, IRCCS Sacro Cuore-Don Calabria, 37024 Negrar, Italy; 5 Pathology Unit, Department of Diagnostics and Public Health, Section of Pathology, University of Verona, 37126 Verona, Italy; 6 ARC-Net Research Center, University of Verona, 37129 Verona, Italy; 7 Pancreatic Surgery Unit, Department of Engineering for Innovation Medicine (DIMI), University of Verona, 37126 Verona, Italy

**Keywords:** duodenal cancer, pancreatic surgery, Whipple procedure, periampullary cancer, survival analysis, lymphadenectomy

## Abstract

**Background/Objectives**: The main treatment for non-ampullary duodenal adenocarcinoma (NDA) is pancreatoduodenectomy (PD) with lymphadenectomy (LN). Several studies have proposed a minimum number of examined lymph nodes (MNELN) to ensure proper staging. This study investigated the impact of nodal parameters—including the pattern of nodal spread—on oncologic outcomes following PD for NDA. Furthermore, we sought to determine the MNELN to ensure reliable detection of nodal involvement. **Methods**: This was a single-center, retrospective study. Consecutive patients who underwent PD from 2000 to 2019 with a final diagnosis of NDA were retrieved from a prospectively maintained database. The probability of detecting at least one metastatic LN in a node-positive patient was assessed using a model based on the binomial probability law. **Results**: A total of 70 patients met the inclusion criteria. The median number of ELNs was 35 (22–43, IQR). Thirty-six patients (51%) had at least one PLN. A node-positive disease was associated with adverse pathologic features, including high tumor grade and perineural and peripancreatic fat invasion. This translated into a greater recurrence rate (*p* < 0.001). The MNELN yielding a 95% probability of detecting at least one metastatic node in a node-positive patient was 25. After a median follow-up of 73 months, the median recurrence-free survival (RFS) was 33 months (95% CI 13–97), and the overall survival (OS) was 41 months (95% CI 17–96). The LN ratio, tumor grade, and metastases at stations 8 and 12 were independently associated with OS (*p* < 0.05). **Conclusions**: Nodal metastases are common among patients with NDA and have a considerable impact on long-term survival. Stations 8 and 12 were associated with OS. Therefore, an adequate lymphadenectomy, possibly including stations 8 and 12, is recommended in patients with NDA.

## 1. Introduction

Non-ampullary duodenal adenocarcinoma (NDA) accounts for 0.5% of all gastrointestinal malignancies and 33–52% of small bowel malignancies (SBAs) [1]. The primary treatment strategy of NDA remains surgical resection with regional lymph node (LN) dissection, although local excision could be considered in case of small lesions. The reported 5-year survival rates following surgical treatment range from 20% to 70% [2]. Several prognostic features have been identified, including TNM staging, tumor site, phenotype, and LN metastases [3]. The number of examined lymph nodes (ELNs) has been shown to affect the oncologic outcomes, in that patients with a greater number of ELNs display longer survival rates, especially within the node-negative class [4]. Improved outcomes with more extensive LN examination, particularly for those patients without known nodal metastasis, suggest that stage migration might contribute to these findings [5]. The extent of LN retrieval is also a significant modifier of the prognostic impact of other node-related parameters, such as the lymph node ratio (LNR) [6]. However, the minimum number of ELNs required for accurate staging in NDA still needs to be better defined. The thresholds proposed by several studies ranged from 5 to 20 nodes [4,5,6,7,8]. Based on these studies, the current National Comprehensive Cancer Network (NCCN) guidelines recommend examining at least eight regional lymph nodes [9]. At the same time, given the paucity of data specific to SBA, information on systemic chemotherapy utilization is extrapolated from colon cancer guidelines, where the minimum number of ELNs for proper staging is 12 [9]. The present study sought to determine the value of nodal staging and the minimum number of ELNs to ensure reliable detection of nodal involvement in pancreatoduodenectomy (PD) for NDA.

## 2. Materials and Methods

This was a single-center, retrospective study. Consecutive patients with NDA who underwent PD from 2000 to 2019 were retrieved from a prospectively maintained institutional database and included in the analysis. Exclusion criteria were endoscopic resection, partial surgical resection (e.g., duodenectomy or local excision), or final pathological diagnosis of ampullary duodenal adenocarcinoma. The preoperative work-up included cross-sectional imaging, laboratory tests, clinical evaluations, and rectal screening for multi-drug-resistant bacteria. The last preoperative imaging was performed at least 4 weeks before surgery. The LN dissection or extension was not influenced by the imaging results (e.g., suspected LN-positive). The institutional protocol for LN dissection included stations 6, 8a-p, 12a-b-c, 13, 14a-v, 17, and the jejunal mesentery nodes. Dissection of stations 7–9–16 was not routinely practiced.

Demographic and clinical details included age, gender, body mass index (BMI), American Society of Anesthesiologists (ASA) score, presence of diabetes, preoperative jaundice, history of familial adenomatous polyposis, celiac disease, Crohn’s disease, weight loss, and neoadjuvant therapy. Postoperative variables included major complications, defined as Clavien–Dindo ≥ III [10]. Pathologic details included R-Status, tumor grading, lymph-vascular, perineural, and peripancreatic fat invasion, number of ELNs, and positive lymph nodes (PLNs). The LNR was determined by dividing the number of PLNs by the number of ELNs and was classified into three categories according to recent literature reports (0 ≤ LNR < 0.2, 0.2 ≤ LNR 0.4, and LNR ≥ 0.4) [7]. All cases were staged according to the American Joint Committee on Cancer, 8th ed. [11]. A pathologist with expertise in gastrointestinal and biliopancreatic cancers analyzed all the specimens (CL). However, the pathological analysis was not standardized according to a formal protocol. Nevertheless, standards of pathological examination were thoroughly discussed between surgeons and pathologists before data analysis and were found to be comparable across the study period. The slicing strategy consisted of bivalve slicing followed by perpendicular slicing (4 mm) [12]. Stations 13 and 17 were examined following orange-peeling and full inclusion in paraffin blocks of peripancreatic soft tissues. Station 14 nodes were examined along the SMA groove. The first jejunal loop mesentery was routinely sampled, and LNs were identified microscopically. Other stations were removed as distinct specimens were fully included and analyzed. The presence of LNs sliced in halves was always specified to avoid double counting. R-Status was defined based on tumor cells within 1 mm from any resection margin. Tumors were staged according to the 8th edition of the AJCC manual from its introduction. For prior resections, tumors were retrospectively restaged from the 7th to the 8th edition.

### Statistical Analysis

Continuous variables were presented as median with interquartile range (IQR) and compared using the Mann–Whitney U-test. Categorical variables were presented as frequencies with percentages and were compared using the Chi-square test or Fisher’s exact test, as appropriate. The probability of detecting at least one metastatic LN in a node-positive patient was assessed using the binomial probability law, as recently described for pancreatic ductal adenocarcinoma [13,14]. Briefly, this probability can be expressed as P = 1 − (1 − *p*)n, where n is the number of ELN and *p* is the global LNR of the study population (total number of PLNs/total number of ELNs), expressing the individual probability for each node to be metastatic. The MNELN was then defined as the cutoff of LN yield associated with a 95% probability of detecting at least 1 PLN in a node-positive patient (P = 95%). Analysis of variables associated with survival was carried out using uni- and multivariable proportional hazard Cox regression models. Survival curves were constructed using the Kaplan–Meier method, and pairwise comparison between groups was performed using the log-rank test. A *p*-value ≤ 0.05 was considered statistically significant. When appropriate, the *p*-values were presented with hazard ratios and 95% confidence intervals (CIs). The data were analyzed using SPSS version 25.0 (IBM Corp., Armonk, NY, USA).

## 3. Results

A total of 70 patients met the inclusion criteria. The median ELN was 35 (IQR 22–43), and 36 patients (51%) had at least one PLN (N+ disease), with a median LNR of 0.26 (IQR 0.10–0.48). Clinical, operative, and pathological characteristics of patients stratified by nodal status are summarized in Table 1. PLNs were more frequently detected in male patients (*p* = 0.048). The presence of a PLN was associated with preoperative jaundice (39% vs. 15%, *p* = 0.021), larger tumor size (median 35 vs. 30 mm, *p* = 0.425), and more advanced T-Status (pT4 69% vs. 21%, *p* < 0.001). Furthermore, the presence of a PLN was associated with other adverse pathologic features, including tumor grade (G2–3 94% vs. 56%, *p* < 0.001), perineural invasion (72% vs. 27%, *p* < 0.001), and pancreatic infiltration (69% vs. 21%, *p* < 0.001). Patients with a PLN received adjuvant treatment, such as chemotherapy, more often (83% vs. 15%, *p* < 0.001). Unsurprisingly, the presence of a PLN was associated with a higher recurrence rate (47% vs. 6%, *p* < 0.001).

**Figure 1:**
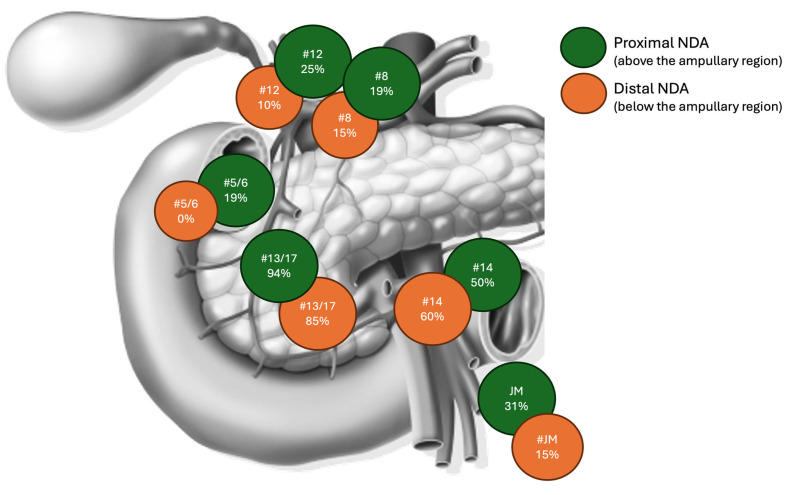
Pattern of nodal spread according to the primary tumor location: proximal (above the ampullary region) vs. distal (below the ampullary region) duodenum.

The pattern of nodal spread is shown in Figure 1, according to the primary tumor location. In tumors arising from the proximal duodenum (above the ampullary region, n = 16), the most commonly involved nodes were at the anterior and posterior surface of the pancreatic head (stations 13–17, 94%). Superior mesenteric artery and vein nodes (station 14a-v) were involved in 50% of patients, jejunal mesentery nodes in 31% of patients, hepatoduodenal ligament nodes (station 12) in 25% of patients, common hepatic artery nodes in 19% of patients, pyloric nodes (stations 5–6) in 19%, and stations 7–9 in 4% of patients. In tumors arising from the distal duodenum (below the ampullary region, n = 16), the most commonly involved nodes were at the anterior and posterior surface of the pancreatic head (stations 13–17, 85%), followed by superior mesenteric artery and vein nodes (station 14a-v, 60%), jejunal mesentery nodes (15%), common hepatic artery nodes (station 8, 15%), and hepatoduodenal ligament nodes (station 12, 10%). Based on the binomial probability law, the MNELN yielding a 95% probability of detecting at least one metastatic node in a node-positive patient was 25.

### 3.1. Overall Survival Analysis

**Figure 2:**
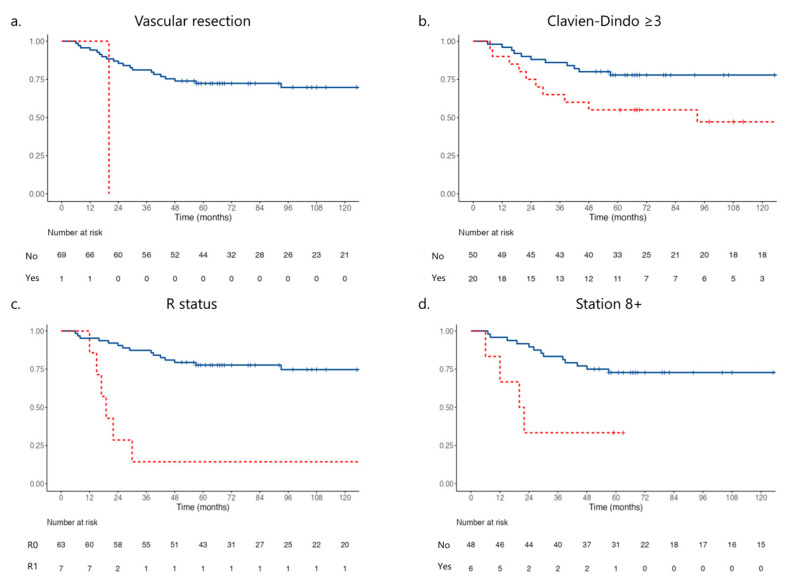
Overall disease-free survival based on the vascular resection ((**a**), blue line no resection, red line vascular resection, *p* = 0.022), Clavien-Dindo (CD) ≥ 3, ((**b**), blue line CD < 2; red line CD ≥ 3, *p* = 0.020), R status ((**c**), blue line R status−, red line R status+, *p* = 0.002) and pathological examination of lymph node station 8, ((**d**), blue line negative nodes, red line positive nodes, *p* = 0.048).

The median follow-up was 73 months. The median overall survival was 41 months (95% CI 17–96). The analysis of clinical, surgical, and pathological factors associated with survival is shown in Table 2 and Table 3. N-Status was correlated to survival (Supplementary Material Figure S1, *p* = 0.002). Vascular resection, severe complications (Clavien-Dindo ≥ 3), R status, and station 8 involvement were independently associated with survival. Kaplan–Meier plots are reported in Figure 2a–d.

### 3.2. Recurrence-Free Survival Analysis

**Figure 3:**
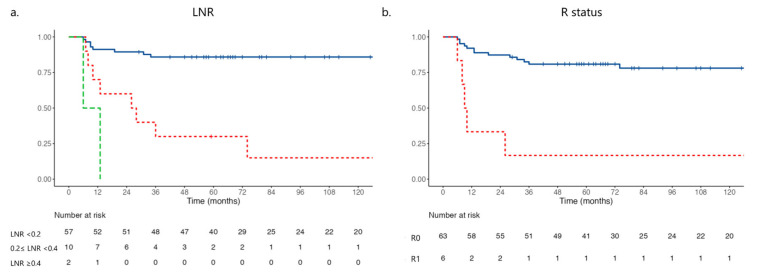
Disease-free survival based on the pathological examination of lymph node ratio ((**a**), blue line LNR < 0.2; red line 0.2 ≤ LNR < 0.4; green line LNR ≥ 0.4, *p* < 0.002), and R status ((**b**), blue line R status−, red line R status+, *p* = 0.021).

The recurrence rate was 27%, with a median recurrence-free survival (RFS) of 33 months (95% CI 13–97). The most common recurrence sites were surgical bed and LN (37%), peritoneum (32%), and liver (21%). Bone, kidney, or brain metastases were less frequent (5%). The analysis of factors associated with DFS is shown in Table 4. N-Status was correlated with survival (Supplementary Material Figure S2, *p* < 0.001). LNR and R-Status were found to be independently associated with DFS (*p* < 0.05). Kaplan–Meier plots are reported in Figure 3a–b.

## 4. Discussion

The number of ELNs has been widely recognized as a determinant of nodal staging after PD, especially for pancreatic ductal adenocarcinoma. Few data are available regarding NDA, and no specific indication of the correct ELN or MNELN is reported.

The present paper investigated the pattern of nodal spread and the MNELN required for optimal staging in NDA. First, nodal metastasis at final pathology was strongly associated with survival. The prognostic role of nodal metastases has been widely described in NDA [7], although the reported data are heterogeneous. The absence of established benchmarks could lead to different surgical and pathological practices, generating a discrepancy in the number of ELNs reported in the literature. This discordance was made more evident by analyzing the different thresholds of LN proposed for a correct NDA staging, ranging from 5 to 16 [4,5,6,7,8,15]. Based on these studies, the National Comprehensive Cancer Network recommended that at least eight regional lymph nodes should be examined for an accurate assessment [9]. A deeper analysis of these surgical series revealed an essential difference in long-term survival with varying ELNs, underlining the crucial prognostic role of nodal staging and the possible bias linked to a stage migration effect [16]. Based on the binomial probability law, the MNELN yielding a 95% probability of detecting at least one metastatic node in a node-positive patient in this series was 25. Importantly, in our study, the median number of ELNs was high (35, IQR 22–43), although, per institutional practice, the lymphadenectomy protocol did not comprise extra-regional nodes (e.g., para-aortic nodes). The surgical and pathological technique standardization led to a correct staging of the patients affected by NDA, reducing the migration stage effect. Indeed, the primary determinants of survival were tumor grading and LNR, and not the presence of nodal metastases, as reported in previous studies [8,15]. The only nodal parameter associated with both recurrence and survival was LNR, with a threshold of 0.4. Several studies investigated the role of LNR following NDA resection [5,7], although with possible biases due to inadequate lymphadenectomy. In the framework of NDA, correct pathologic staging is of the utmost importance because local recurrence in the LN is common.

In addition to the MNELN and the LNR, the present study investigated the pattern of nodal spread. Regardless of tumor site (proximal or distal duodenum), nodes at the anterior and posterior surface of the pancreatic head were most commonly metastatic, followed by nodes along the superior mesenteric artery. This is consistent with a recent analysis of 36 patients who underwent PD at a Japanese center [17]. However, from a prognostic standpoint, only the involvement of common hepatic artery nodes and nodes in the hepatoduodenal ligament was associated with reduced recurrence-free and overall survival. Therefore, nodal dissection for NDA should routinely entail these stations.

The study has some limitations. First, the retrospective design could generate a bias. Second, even if NDA is a rare entity, the relatively small sample size could be a limitation. Third, our nodal dissection protocol may not stick with the common practice of nodal retrieval in PD. Indeed, different authors do not routinely dissect the common hepatic artery and the hepatoduodenal ligament node stations.

## 5. Conclusions

In conclusion, LN metastases are common in patients with NDA and have a considerable impact on long-term survival. The pattern of nodal spread is similar to other periampullary cancers. The hepatoduodenal ligament and hepatic artery lymph node stations were associated with disease recurrence and survival. Therefore, PD and appropriate LN harvesting should be carried out to stage patients with NDA properly.

## Figures and Tables

**Table 1:** Clinical, operative, and pathological characteristics of patients undergoing pancreaticoduodenectomy for non-ampullary duodenal carcinoma, stratified by nodal status.

	Node Negative (n = 34, 49%)	Node Positive (n = 36, 51%)	*p*-Value
Baseline Parameters			
Female, n (%)	17 (50%)	10 (28%)	**0.048**
Age > 65 y, n (%)	13 (38%)	13 (36%)	0.525
BMI > 25 kg/m^2^, n (%)	11 (32%)	14 (39%)	0.375
ASA ≥ 3, n (%)	4 (12%)	7 (19%)	0.291
Diabetes, n (%)	0 (0%)	4 (11%)	0.064
Preoperative Jaundice, n (%)	5 (15%)	14 (39%)	**0.021**
History of Malignancy, n (%)	9 (27%)	5 (14%)	0.155
Neoadjuvant Therapy, n (%)	4 (12%)	0 (0%)	0.051
Intraoperative Parameters			
Whipple Resection, n (%)	17 (50%)	14 (39%)	0.244
Multiorgan Resection, n (%)	1 (3%)	3 (8%)	0.329
Vascular Resection, n (%)	0 (0%)	1 (3%)	0.514
EBL > 400 mL, n (%)	15 (44%)	19 (53%)	0.314
OT > 360 min, n (%)	23 (68%)	20 (56%)	0.214
Pathological Parameters			
Intestinal Phenotype	26 (77)	23 (64)	0.188
Tumor Size (mm), median (IQR)	30 (0–75)	35 (0–160)	0.425
Location, n (%)			0.146
Oral	10 (29%)	16 (44%)	
Anal	24 (71%)	20 (56%)	
ELN, median (IQR)	30 (21–43)	38 (31–46)	**0.032**
pT, n° (%)			**<0.001**
1	15 (44%)	0 (0%)	
2	3 (9%)	3 (8%)	
3	9 (27%)	8 (22%)	
4	7 (21%)	25 (69%)	
Grading, n (%)			**<0.001**
Well Differentiated	15 (44%)	2 (6%)	
Moderately differentiated	11 (32%)	22 (61%)	
Poorly Differentiated	8 (24%)	12(33%)	
R1, n (%)	2 (6%)	5 (14%)	0.239
Pancreatic Infiltration, n (%)	7 (21%)	25 (69%)	**<0.001**
Perineural Invasion, n (%)	9 (27%)	26 (72%)	**<0.001**
Adjuvant Therapy	5 (15%)	30 (83%)	**<0.001**
Recurrence	2 (6%)	17 (47%)	**<0.001**

Bold values indicate statistical significance (*p* < 0.05); BMI: body mass index; ASA: American Society of Anesthesiology; OT: operation time; EBL: estimated blood loss; ELN: estimated lymph node.

**Table 2:** Cox regression model of clinical and surgical survival predictors.

Variables	Univariate Hazard Ratio (95% CI)	*p*-Value	Multivariate Hazard Ratio (95% CI)	*p*-Value
Baseline Parameters				
Female	0.394 (0.145–1.071)	0.068		
Age > 65 y	1.941 (0.839–4.486)	0.121		
BMI > 25 kg/m^2^	1.631 (0.702–3.793)	0.255		
ASA Score ≥ 3	1.983 (0.720–5.461)	0.185		
Diabetes	2.375 (0.549–10.268)	0.247		
Preoperative Jaundice	1.035 (0.404–2.651)	0.943		
History of Other Malignancies	0.880 (0.298–2.601)	0.817		
Neoadjuvant Therapy	0.680 (0.091–5.082)	0.707		
Intraoperative Parameters				
Whipple Resection	0.626 (0.262–1.496)	0.292		
Multiorgan Resection	0.810 (0.109–6.037)	0.837		
Vascular Resection	8.114 (1.015–64.881)	**0.048**	12.070 (1.44–101.11)	**0.022**
OT > 360 min	1.115 (0.467–2.662)	0.806		
EBL > 400 mL	1.165 (0.504–2.690)	0.721		
Postoperative Parameters				
Postoperative Complications	2.172 (0.634–7.438)	0.217		
Clavien–Dindo ≥ III	2.538 (1.094–5.892)	**0.030**	2.766 (1.171–6.533)	**0.020**

Bold values indicate statistical significance (*p* < 0.05); BMI: body mass index; ASA: American Society of Anesthesiology; OT: operation time; EBL: estimated blood loss.

**Table 3:** Cox regression model of pathological survival predictors.

Variables	Univariate Hazard Ratio (95% CI)	*p*-Value	Multivariate Hazard Ratio (95% CI)	*p*-Value
Tumor Size	0.999 (0.983–1.014)	0.855		
pT3–4	3.395 (1.003–11.493)	**0.050**	-	0.546
pN+	4.359 (1.597–11.900)	**0.004**	-	0.281
ELN > 25	2.695 (0.908–7.998)	0.074		
0.2 ≤ LNR < 0.4	4.648 (1.821–11.867)	**0.001**	-	0.157
LNR ≥ 0.4	6.863 (1.520–30.994)	**0.012**	-	0.424
Grading (G2–G3)	4.278 (0.992–18.450)	**0.002**	-	0.455
Location Anal	0.639 (0.275–1.480)	0.296		
Non-Intestinal Phenotype	2.120 (0.901–4.987)	0.085		
R-Status	7.241 (2.724–19.246)	**<0.001**	5.994 (1.906–18.847)	**0.002**
Lymph-vascular invasion	4.543 (1.341–15.386)	**0.015**	-	0.718
Perineural Invasion	2.614 (1.063–6.426)	**0.036**	-	0.941
Pancreatic Infiltration	2.795 (1.161–6.730)	**0.022**	-	0.910
Adjuvant Therapy	2.547 (1.037–6.254)	**0.041**	-	0.535
Lymph Nodes Station Sub-Analysis				
Positive Station 14	4.677 (1.870–11.700)	**<0.001**	-	0.514
Positive Stations 13–17	5.085 (1.853–13.957)	**0.002**	-	0.563
Positive Station 12 a, b, c	7.783 (2.756–21.975)	**<0.001**	-	0.307
Positive Stations 5–6	3.776 (1.194–11.944)	**0.024**	-	0.507
Positive Stations 7–9	4.763 (0.766–29.619)	0.094		
Positive Station 8 a, p	4.367 (1.406–13.564)	**0.011**	8.076 (1.015–64.25)	**0.048**
Positive Station 16	-	-		
Positive Station Jejunal Mesentery	10.005 (3.710–26.981)	**<0.001**	-	-

Bold values indicate statistical significance (*p* < 0.05); ELN: estimated lymph node; LNR: lymph node ratio.

**Table 4:** Cox regression model of pathological disease-free survival predictors.

Variables	Univariate Hazard Ratio (95% CI)	*p*-Value	Multivariate Hazard Ratio (95% CI)	*p*-Value
Tumor Size	0.966 (0.979–1.014)	0.688		
pT3–4	4.117 (0.946–17.915)	0.059		
pN+	9.550 (2.192–41.603)	**0.003**	-	0.451
ELN > 25	4.580 (1.050–19.973)	**0.043**	-	0.065
0.2 ≤ LNR < 0.4	8.090 (3.003–21.795)	**<0.001**	-	0.105
LNR ≥ 0.4	23.891 (4.652–122.691)	**<0.001**	53.537 (4.563–623.886)	**0.002**
Grading (G2–3)	6.707 (0.892–50.455)	0.065		
Location Anal	0.678 (0.267–1.719)	0.412		
Non-Intestinal Phenotype	2.934 (1.158–7.433)	**0.023**	-	0.923
R-Status	8.127 (2.810–23.502)	**<0.001**	5.946 (1.315–26.874)	**0.021**
Lymph-vascular invasion	11.781 (1.567–88.589)	**0.017**	-	0.374
Perineural Invasion	3.042 (1.083–8.545)	**0.035**	-	0.937
Pancreatic Infiltration	3.808 (1.354–10.706)	**0.011**	-	0.796
Adjuvant Therapy	2.231 (0.837–5.946)	0.109		
Lymph Nodes Station Sub-Analysis				
Positive Station 14	10.494 (3.352–32.855)	**<0.001**	-	0.059
Positive Stations 13–17	11.245 (2.566–49.271)	**0.001**	-	0.130
Positive Station 12 a, b, c	9.216 (3.123–27.197)	**<0.001**	-	0.905
Positive Stations 5–6	2.136 (0.466–9.788)	0.328		
Positive Stations 7–9	4.763 (0.766–29.619)	0.094		
Positive Station 8 a, p	7.908 (2.694–23.831)	**<0.001**	-	0.102
Positive Station 16	5.660 (0.513–62.450)	0.157		
Positive Station 15	9.606 (3.499–26.369)	**<0.001**	-	0.680

Bold values indicate statistical significance (*p* < 0.05); ELN: estimated lymph node; LNR: lymph node ratio.

## Data Availability

The data presented in this study are available on request from the corresponding author.

## Supplementary Materials

The following supporting information can be downloaded at: https://www.mdpi.com/article/10.3390/jcm14082616/s1, Figure S1: Overall Survival based on N-Status (*p* = 0.002); Figure S2: Overall Disease-Free Survival based on N-Status (*p* = 0.002).

## References

[1] Locher C., Batumona B., Afchain P., Carrère N., Samalin E., Cellier C., Aparicio T., Becouarn Y., Bedenne L., Michel P. (2018). Small bowel adenocarcinoma: French intergroup clinical practice guidelines for diagnosis, treatments, and follow-up (SNFGE, FFCD, GERCOR, UNICANCER, SFCD, SFED, SFRO). Dig. Liver Dis..

[2] Hirashita T., Ohta M., Tada K., Saga K., Takayama H., Endo Y., Uchida H., Iwashita Y., Inomata M. (2018). Prognostic factors of non-ampullary duodenal adenocarcinoma. Jpn. J. Clin. Oncol..

[3] Struck A., Howard T., Chiorean E.G., Clarke J.M., Riffenburgh R., Cardenes H.R. (2009). Non-ampullary duodenal adenocarcinoma: Factors important for relapse and survival. J. Surg. Oncol..

[4] Thiessen M., Lee-Ying R.M., Monzon J.G., Tang P.A. (2020). An Examination of Lymph Node Sampling as a Predictor of Survival in Resected Node-Negative Small Bowel Adenocarcinoma: A SEER Database Analysis. J. Gastrointest. Cancer.

[5] Ecker B.L., McMillan M.T., Datta J., Dempsey D.T., Karakousis G.C., Fraker D.L., Drebin J.A., Mamtani R., Giantonio B.J., Roses R.E. (2016). Lymph node evaluation and survival after curative-intent resection of duodenal adenocarcinoma: A matched cohort study. Eur. J. Cancer Oxf. Engl..

[6] Yoshimizu S., Kawachi H., Yamamoto Y., Nakano K., Horiuchi Y., Ishiyama A., Tsuchida T., Yoshio T., Hirasawa T., Ito H. (2020). Clinicopathological features and risk factors for lymph node metastasis in early-stage non-ampullary duodenal adenocarcinoma. J. Gastroenterol..

[7] Tran T.B., Qadan M., Dua M.M., Norton J.A., Poultsides G.A., Visser B.C. (2015). Prognostic relevance of lymph node ratio and total lymph node count for small bowel adenocarcinoma. Surgery.

[8] Zheng L. (2020). Negative Lymph Node Count is an Independent Impact Factor for Predicting the Specific Survival of Primary Duodenal Neoplasms under Surgical Procedures. Clin. Lab..

[9] Benson A.B., Venook A.P., Al-Hawary M.M., Arain M.A., Chen Y.-J., Ciombor K.K., Cohen S.A., Cooper H.S., Deming D.A., Garrido-Laguna I. (2019). Small Bowel Adenocarcinoma, Version 1.2020, NCCN Clinical Practice Guidelines in Oncology. J. Natl. Compr. Cancer Netw. JNCCN.

[10] Dindo D., Demartines N., Clavien P.-A. (2004). Classification of Surgical Complications. Ann. Surg..

[11] Amin M.B., Edge S., Greene F., Byrd D.R., Brookland R.K., Washington M.K., Gershenwald J.E., Compton C.C., Hess K.R., Sullivan D.C. (2017). AJCC Cancer Staging Manual.

[12] Adsay N.V., Basturk O., Altinel D., Khanani F., Coban I., Weaver D.W., Kooby D.A., Sarmiento J.M., Staley C. (2009). The number of lymph nodes identified in a simple pancreatoduodenectomy specimen: Comparison of conventional vs orange-peeling approach in pathologic assessment. Mod. Pathol..

[13] Vuarnesson H., Lupinacci R.M., Semoun O., Svrcek M., Julié C., Balladur P., Penna C., Bachet J.B., Resche-Rigon M., Paye F. (2013). Number of examined lymph nodes and nodal status assessment in pancreaticoduodenectomy for pancreatic adenocarcinoma. Eur. J. Surg. Oncol..

[14] Malleo G., Maggino L., Ferrone C.R., Marchegiani G., Mino-Kenudson M., Capelli P., Rusev B., Lillemoe K.D., Bassi C., Fernàndez-del Castillo C. (2019). Number of Examined Lymph Nodes and Nodal Status Assessment in Distal Pancreatectomy for Body/Tail Ductal Adenocarcinoma. Ann. Surg..

[15] Sakamoto T., Saiura A., Ono Y., Mise Y., Inoue Y., Ishizawa T., Takahashi Y., Ito H. (2017). Optimal Lymphadenectomy for Duodenal Adenocarcinoma: Does the Number Alone Matter?. Ann. Surg. Oncol..

[16] Sarela A.I., Brennan M.F., Karpeh M.S., Klimstra D., Conlon K.C.P. (2004). Adenocarcinoma of the duodenum: Importance of accurate lymph node staging and similarity in outcome to gastric cancer. Ann. Surg. Oncol..

[17] Nishio K., Kimura K., Eguchi S., Shirai D., Tauchi J., Kinoshita M., Murata A., Ohira G., Shinkawa H., Shintaro K. (2022). Prognostic Factors and Lymph Node Metastasis Patterns of Primary Duodenal Cancer. World J. Surg..

