# Supplementary material for: Positive Lymph Nodes Independently Affect Long-Term Survival After Pancreaticoduodenectomy for Non-Ampullary Duodenal Adenocarcinoma: A Single-Center, Retrospective Analysis
Source: J Clin Med. 2025 Apr 11;14(8):2616. doi: 10.3390/jcm14082616 (PMC12028301; doi:10.3390/jcm14082616)
Supplement: Supplementary file 1 [file jcm-14-02616-s001.zip › jcm-3491565-supplementary.pdf]

Supplementary Material Figure S1. Overall Survival based on N-Status ($p = 0.002$).

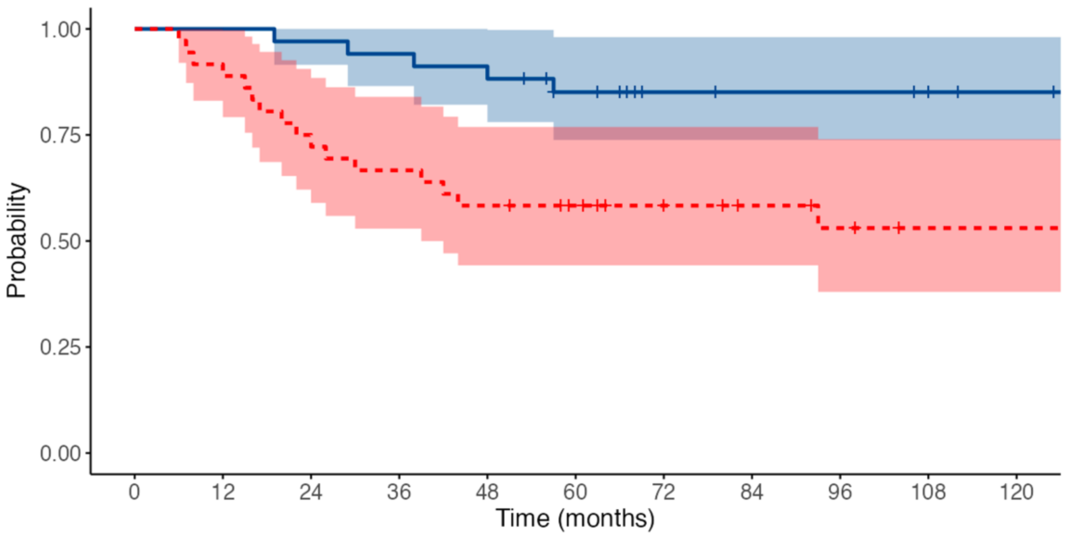

Number at risk

N0	34	34	33	32	31	26	17	16	16	15	13
N+	36	33	27	24	21	18	15	12	10	8	8

Supplementary Material Figure S2. Overall Disease-Free Survival based on N-Status ($p < 0.001$).

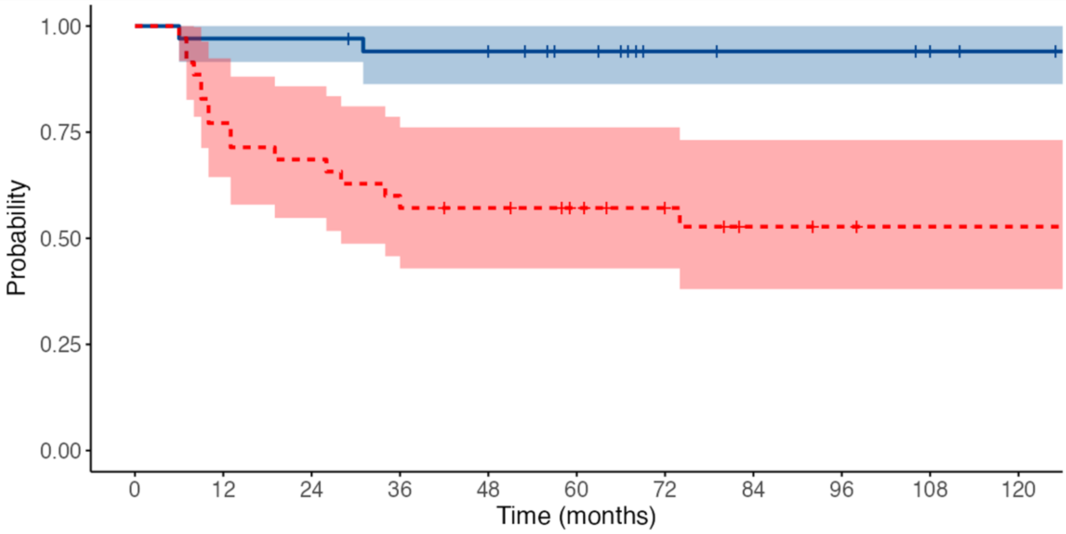

Number at risk

N0	34	33	33	31	31	26	17	16	16	15	13
N+	35	27	24	21	19	16	14	10	9	8	8
